# Blockage of Notch Signaling Inhibits the Migration and Proliferation of Retinal Pigment Epithelial Cells

**DOI:** 10.1155/2013/178708

**Published:** 2013-12-25

**Authors:** Weiwei Liu, Guorong Jin, Chongde Long, Xin Zhou, Yan Tang, Shan Huang, Xielan Kuang, Lizi Wu, Qingjiong Zhang, Huangxuan Shen

**Affiliations:** ^1^State Key Laboratory of Ophthalmology, Zhongshan Ophthalmic Center, Sun Yat-sen University, 54 Xianlie Road, Guangzhou 510060, China; ^2^Institute of Cancer Stem Cell, Dalian Medical University, Dalian 116023, China; ^3^Biobank of Eye, State Key Laboratory of Ophthalmology, Zhongshan Ophthalmic Center, Sun Yat-sen University, 54 Xianlie Road, Guangzhou 510060, China; ^4^Department of Molecular Genetics and Microbiology, Shands Cancer Center, University of Florida, Gainesville, FL 32610, USA

## Abstract

The Notch signaling is an evolutionarily conserved cell-cell communication pathway that plays critical roles in the proliferation, survival, apoptosis, and fate determination of mammalian cells. Retinal pigment epithelial (RPE) cells are responsible for supporting the function of the neural retina and maintaining vision. This study investigated the function of Notch signaling in RPE cells. We found that the members of the Notch signaling pathway components were differentially expressed in RPE cells. Furthermore, blockage of Notch signaling inhibited the migration and proliferation of RPE cells and reduced the expression levels of certain Notch signaling target genes, including HES1, MYC, HEY2, and SOX9. Our data reveal a critical role of Notch signaling in RPE cells, suggesting that targeting Notch signaling may provide a novel approach for the treatment of ophthalmic diseases related to RPE cells.

## 1. Introduction

The retinal pigment epithelium (RPE), a polarized monolayer of specialized epithelium and a glial cell layer located between the neural retina and choroid, plays a pivotal role in supporting photoreceptor function [1, 2]. RPE cells function as the guardians and caretakers of the photoreceptors. These cells protect the photoreceptors from photooxidation, phagocytose shed photoreceptor membranes, and transport water and nutrients, such as glucose, retinol, and fatty acids [3].

Notch signaling is an evolutionarily conserved cell-cell communication mechanism that regulates the proliferation, survival, apoptosis, and fate decision-making of mammalian cells through local cell interactions [4]. Notch signaling is activated by the interaction between Notch transmembrane receptors and a family of plasma membrane-associated ligands. In vertebrates, the Notch signaling pathway includes four receptors (Notch1-4) and five ligands (Delta1, Delta3, Delta4, Jagged1, and Jagged2) [5]. The Notch genes are conserved, with four extremely similar receptors exhibiting subtle differences in certain domains.

Notch signaling is activated by a ligand binding to the receptor between two neighboring cells [6]. Upon ligand binding, the Notch receptors undergo multiple proteolytic cleavages, leading to the release of the Notch intracellular domain (NICD), which subsequently translocates into the nucleus. In the nucleus, NICD forms a transcriptional complex that activates the Notch target genes, including the HES family, the HEY family, and the ID family [7]. Notch signaling affects cell proliferation, survival, apoptosis, and fate determination by regulating the expression of target genes.

To date, few reports clarify the role of Notch signaling in human RPE cells. In this study, we examined the effect of Notch signaling on the migration and proliferation of RPE cells and the expression levels of Notch target genes after inhibiting Notch signaling by knocking down NOTCH1. Our study revealed a role of Notch signaling in regulating the proliferation and migration of human RPE cells, which is important in understanding the pathogenesis of certain ophthalmic diseases.

## 2. Materials and Methods

### 2.1. Plasmids, Small Hairpin RNAs, and Reagents

The following small hairpin RNA (shRNA) lentiviral constructs targeting the human NOTCH1 gene were obtained from Thermo Scientific and have been previously described [8]. The hairpin sequence numbers are TRCN0000003358-61.

The following antibodies were used in Western blotting: NOTCH1 (sc-6014R, Santa Cruz Biotechnology Inc., Santa Cruz, CA, 1 : 250), GAPDH (20120829, Zen BioScience, Chengdu, China, 1 : 1000) [8].

### 2.2. Cell Culture and Lentiviral Transduction

The ARPE-19 cells (ATCC) were cultured in DMEM with 10% inactivated fetal calf serum (FCS), penicillin (100 units/mL), and streptomycin (50 *μ*g/mL) at 37°C in a 5% CO_2_ atmosphere.

Lentiviral transduction was performed as previously described [9]. In brief, lentiviral vectors targeting NOTCH1, together with the packing plasmid PSPAX2 and pseudotyped envelope pMD2.G, were transfected into 293T cells using the Effectene Transfection Reagent (Qiagen). RPE cells were plated at 50–70% confluence in 60 mm plates and were subsequently infected four times with 1.5 mL of virus plus 0.5 mL of fresh complete medium containing 8 *μ*g/mL Polybrene (Sigma-Aldrich). Finally, the RPE cells were selected with puromycin (1.6 *μ*g/mL, Sigma) for one week.

### 2.3. Proliferation and Scratch Assays

Proliferation and scratch assays were performed as previously described [10]. For the proliferation assay, RPE cells (6 × 10^4^) were cultured in 24-well plates. Cells were then trypsinized, and the number of cells was determined by trypan blue assays on the following 4 days. For the scratch assay, RPE cells were seeded into a 6-well plate to reach 80–90% confluence. A scratch was made using a white tip and the cells were cultured in DMEM medium without fetal bovine serum. Migration of the cells into the scratch area was observed at 12, 24, and 36 hours after the scratch had been made.

### 2.4. Real-Time RT-PCR and Western Blotting

For real-time RT-PCR, total RNA was extracted from RPE cells using the Trizol reagent (Invitrogen), and cDNA was reverse transcribed using the PrimeScript II 1st Strand cDNA Synthesis Kit (TaKaRa). Real-time RT-PCR analysis was performed on an ABI PRISM 7000 Sequence Detector using the SYBR Premix Ex TaqTM Kit (TaKaRa). The sequences of the 14 pairs of primers used are listed in Supplementary Tables 1 and 2 of the Supplementary Material availbale online at http://dx.doi.org/10.1155/2013/178708.

For Western blotting, total protein was extracted using lysis buffer. Ninety micrograms of protein was separated by electrophoresis using 7.5% SDS-polyacrylamide gels. The details of Western blotting were as described previously [11].

## 3. Results

### 3.1. RPE Cells Expressed Different Levels of Notch Signal Pathway Components

**Figure 1:**
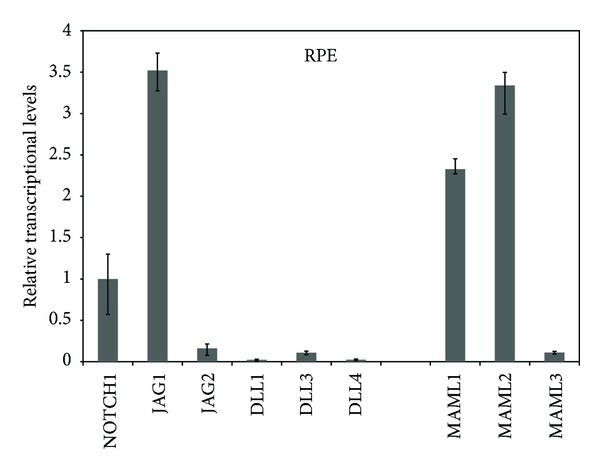
The members of the Notch signaling pathway components were differentially expressed in RPE cells. The relative expression levels of Notch signaling components in RPE cells were determined by real-time RT-PCR analysis. The detected components included five Notch ligands (JAG1, JAG2, DLL1, DLL3, and DLL4) and three MAML transcriptional coactivators (MAML1, MAML2, and MAML3). Error bars represent at least two independent experiments.

We performed real-time RT-PCR to determine the expression levels of the Notch signaling pathway components, including five Notch ligands (JAG1, JAG2, DLL1, DLL3, and DLL4) and three MAML transcriptional coactivators (MAML1, MAML2, and MAML3). We found that JAG1 had a higher expression level than any of the other ligands (JAG2, DLL1, DLL3, and DLL4). MAML1 and MAML2 were expressed at significant levels, with MAML3 displaying the lowest expression level (Figure 1).

### 3.2. Inhibition of Notch Signaling by Knocking Down NOTCH1 Using Several shRNAs

**Figure 2:**
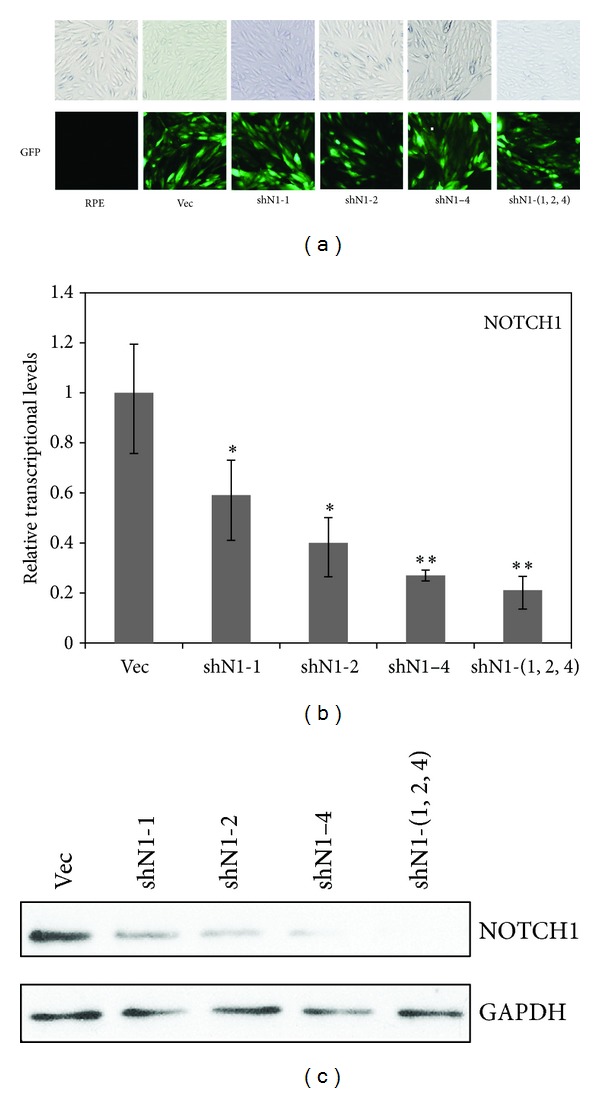
Notch signaling was blocked by lentiviral-based small hairpin RNA (shRNA) targeting NOTCH1. (a) Cell lines in which NOTCH1 was inhibited were established using the lentiviral system. Cells were successfully infected with shNOTCH1-lentivirus expressing GFP, which can be observed under a fluorescence microscope. (b) The knockdown efficiency of NOTCH1 was determined by real-time RT-PCR analysis. (c) The downregulation of NOTCH1 proteins was detected by Western blot analysis using GAPDH as an internal control. Data are expressed as means ± SD. *P < 0.05 versus vehicle and error bars represent at least two independent experiments.

To determine the role of Notch signaling in RPE cells, we knocked down the expression of NOTCH1 using several NOTCH1-shRNAs. We chose three lentiviral vectors targeting NOTCH1 (shN1-1, shN1-2, and shN1-4). After the RPE cells were selected with puromycin, we obtained four stable RPE cell lines with different levels of NOTCH1 knockdown. The shNOTCH1-(1, 2, 4) was the cell line that was infected with a mixture of the three lentiviruses, including shN1-1, shN1-2, and shN1-4 (Figure 2(a)). Next, we determined the efficiency of NOTCH1 knockdown using real-time RT-PCR (Figure 2(b)) and Western blotting (Figure 2(c)), and we observed that the mRNA and protein levels of NOTCH1 were both reduced in the four groups of shNOTCH1-transduced RPE cells. The combination of the three shNOTCH1 viruses (shN1-1, 2, 4) resulted in the most significant NOTCH1 knockdown (about 90%). Thus, we obtained stable NOTCH1-knockdown RPE cells.

### 3.3. Blockage of Notch Signal Pathway Inhibited the Migration and Proliferation of RPE Cells

**Figure 3:**
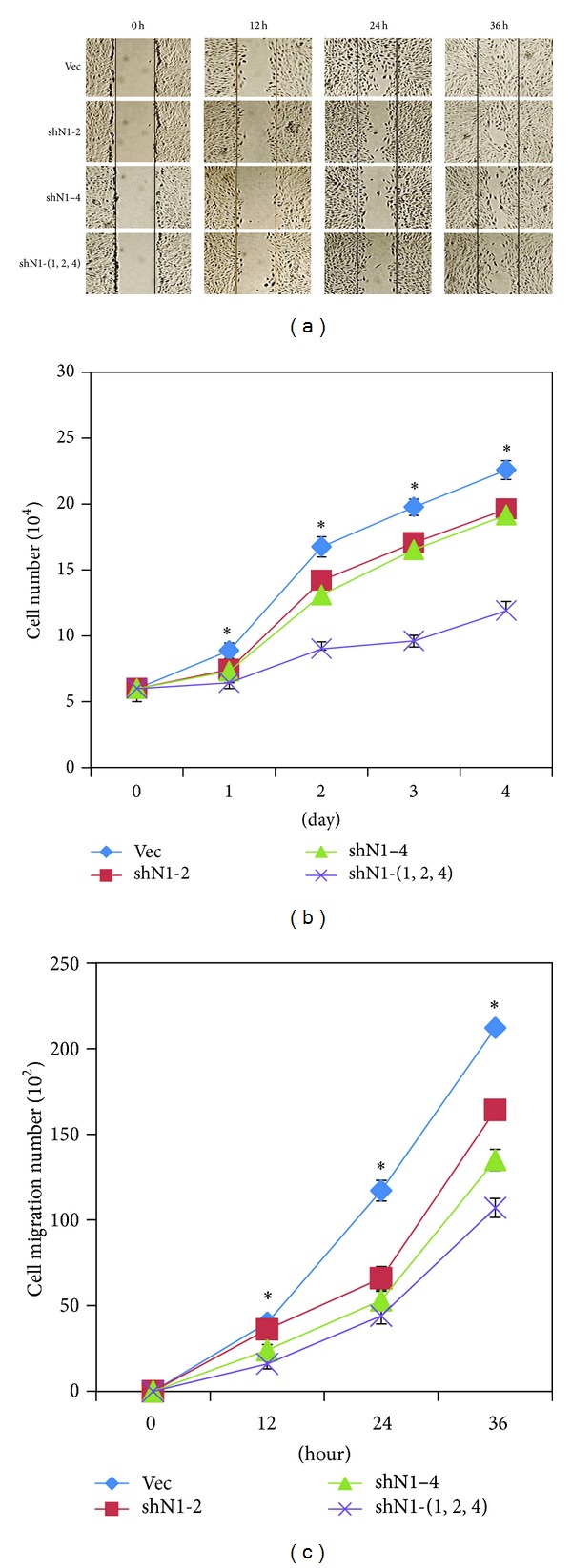
Knockdown of NOTCH1 inhibited the migration and proliferation of RPE cells. (a) The migratory ability of RPE cells was analyzed using the scratch assay. The migration of RPE cell lines was inhibited after NOTCH1 was knocked down (24 h and 36 h). (b) Cells that migrated to the scratch wound were counted. The cell line shN1-(1, 2, 4) exhibited the lowest number of cells that migrated to the scratch area when compared to control cells. (c) RPE cell proliferation was determined by cell counting for four days. Data are presented as average values ± SD. The cell line shN1-(1, 2, 4) showed the slowest proliferation rate. Data are expressed as means ± SD. *P < 0.05 versus vehicle and error bars represent at least two independent experiments.

To determine whether blockage of Notch signaling has an effect on RPE cell migration, we monitored the migratory capacities of RPE cells using the *in vitro* scratch assay. The control cells displayed high migratory ability, as the scratch wound was almost recovered after 36 h of incubation. However, the migration of the three NOTCH1-knockdown RPE cell lines was significantly inhibited (Figure 3(a)). RPE cells that were infected with the mixture of the three shNOTCH1 viruses exhibited the lowest number of cells that migrated to the scratch area (Figure 3(b)).

In addition, we investigated the effect of blocking Notch signaling on RPE cell proliferation. After inhibiting the Notch signaling pathway, a reduction of RPE cell proliferation was detected. As in the scratch assay, the most significant decrease in cell growth was observed in the shN1-(1, 2, 4) cells (Figure 3(c)). Therefore, blockage of Notch signaling via NOTCH1 knockdown reduces the migration and proliferation of RPE cells.

### 3.4. Inhibition of Notch Signaling Reduced the Expression of Several Notch Signaling Target Genes

**Figure 4:**
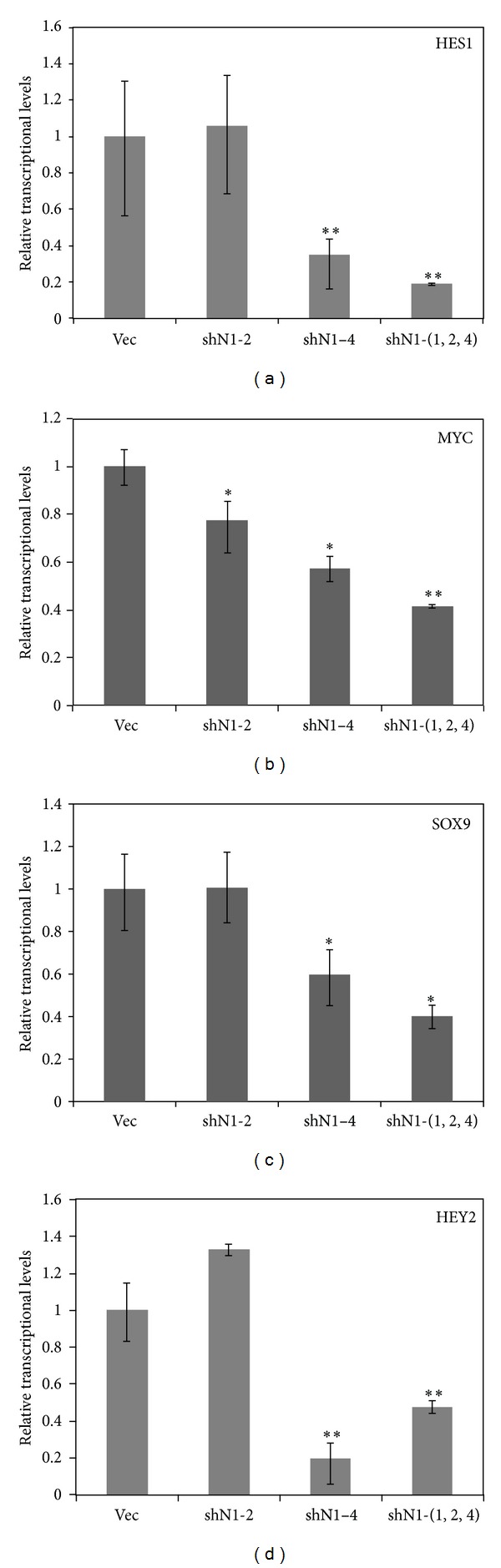
Inhibition of Notch signaling reduced the expression of the Notch signaling target genes. The downregulation of four members of the Notch signaling pathway (HES1, MYC, HEY2, and SOX9) was determined using real-time RT-PCR. Data are expressed as means ± SD. *P < 0.05 versus vehicle and error bars represent at least two independent experiments.

To investigate the mechanism of the effects of blocking the Notch signaling on the migration and proliferation of RPE cells, we performed real-time RT-PCR to follow the expression levels of Notch signaling target genes. Among the twelve target genes we selected, four genes, including HES1, SOX9, HEY2, and MYC, exhibited significant downregulation after NOTCH1 was knocked down (Figure 4).

## 4. Discussion

The RPE is a single layer of epithelial cells located at the posterior segment of the eye and is crucial for photoreceptor function. The RPE is relevant to several photoreceptor diseases, such as proliferative vitreoretinopathy (PVR) [12], proliferative diabetic retinopathy (PDR) [13], and age-related macular degeneration (AMD) [14]. In these diseases, RPE cells exhibit disordered proliferation and migration.

Different activation levels of Notch signaling induce distinct dose-dependent phenotypes. More specifically, high levels of Notch signaling activation cause a suppression of cell proliferation and downregulation of the expression of certain matrix-adhesion molecules. However, lower levels of Notch signaling activation lead to a proliferative response and maintain matrix adhesion in mammary epithelial cells [15]. In this study, we inhibited the Notch signaling to investigate its role in PRE and discussed the relationship between Notch signaling and several photoreceptor diseases.

To establish the stable RPE cell line with inhibited Notch signaling, we knocked down NOTCH1 using several shRNAs. The efficiency of knockdowns was different, and the most effective knockdown was achieved with the mixture of viruses (shN1-1, 2, 4) (Figures 2(b) and 2(c)). Consistent with the efficiency of interference, the cell line shN1-(1, 2, 4) had the slowest rate of migration and proliferation (Figure 3). Another group has reported that the constitutive activity of Notch in transgenic mice expressing the intracellular domain of the Notch1 leads to a hyperproliferation of RPE cells [16]. In this study, our results indicated that inhibition of Notch signaling *in vitro* reduced the migration and proliferation of RPE cells. In ophthalmic diseases, such as PVR and AMD, the RPE can initiate disordered migration and proliferation [17]. Therefore, the abnormal migration and proliferation of RPE cells in these diseases might be related to the dysregulated Notch signaling.

To explore the mechanism by which a blockage in Notch signaling reduced migration and proliferation in RPE cells, we performed real-time RT-PCR to evaluate the expression levels of several critical transcription factors involved in Notch signaling. The HES family is a family of transcriptional repressors that act as Notch effectors [18], and its member HES1 is essential in regulating mammalian neuronal differentiation [19]. MYC is also a direct target of Notch signaling, functioning as a transcription factor that regulates the transcription of specific target genes [20]. SOX9 is a transcription factor that belongs to the SOXE group of the sex-determining region-related HMG-box family, and SOX9 plays a crucial role in the development of many biological processes. HEY2 encodes a member of the enhancer of split-related (HESR) family of transcription factors, the expression of which is induced by Notch signaling. Our data indicated that blockage of Notch signaling significantly repressed the expression levels of these four target genes. In particular, others have reported that lacking HEY2 decreased the proliferation and migration of cultured vascular smooth muscle cells [21]. Therefore, inhibition of the migration and proliferation after blocking Notch signaling in RPE cells might be related to a reduction in HEY2 expression levels. HEY2 might have a synergistic role in the regulation of the inhibition and migration in RPE cells with other target genes, such as HES1, MYC, and SOX9.

## 5. Conclusion

To the best of our knowledge, few reports clarify the role of Notch signaling in human RPE cells. We showed that RPE cell lines expressed different levels of Notch signaling components. Our results also highlighted the role of Notch signaling in regulating the migration and proliferation of RPE cells. In addition, the change in Notch signaling altered the expression levels of Notch signaling target genes that might participate in the regulation of migration and proliferation in RPE cells. Our results provide a rational background for understanding Notch signaling and RPE cells more comprehensively and suggest that focusing on the Notch signaling and the Notch target genes may provide a novel approach for the treatment of ophthalmic diseases related to RPE cells.

## Supplementary Material

Table 1:The primers of Notch signaling components used in real time RT-PCR.
Table 2: The primers of Notch signaling target genes used in real-time RT-PCR.
