# Supplementary material for: Blockage of Notch Signaling Inhibits the Migration and Proliferation of Retinal Pigment Epithelial Cells
Source: ScientificWorldJournal. 2013 Dec 25;2013:178708. doi: 10.1155/2013/178708 (PMC3885266; doi:10.1155/2013/178708)
Supplement: Supplementary file 1 — Table 1:The primers of Notch signaling components used in real time RT-PCR. Table 2: The primers of Notch signaling target genes used in real-time RT-PCR. [file 178708.f1.pdf]

Supplementary

TABLE1 The primers of Notch signaling components used in real time RT-PCR

NOTCH1-F	GATGACCTGGGCAAGTCCG
NOTCH1-R	TGCGCTCCTGTGCGATGT
JAG1-F	ACGACCCCCTGTGAAGTGAT
JAG1-R	TCCCGACTGACTCTTGCACT
JAG2-F	CGCCAATGAGTGTGAAGGGA
JAG2-R	TCGTTGACGTTGATATGGCAGT
DLL1-F	ACCTCGCAACAGAAAACCCA
DLL1-R	GTGTTCGTCACACACGAAGC
DLL3-F	GGTCCGAGCTCGTCCGTAG
DLL3-R	GAAAAGGGGCGTCGCTACC
DLL4-F	ATCAGCGATATGCTCCCCCA
DLL4-R	TGCCTTATACCTCCGTGGCA
MAML1-F	GCCACGCATCTTCATGATACAG
MAML1-R	CCATTGGAAGAGATGGCAACTC
MAML2-F	CTGATTGCGCTCCAGGGTTC
MAML2-R	TCCACAAAGCCATTGGGTCG
MAML3-F	ACCACACGCTGATCATGCTAC
MAML3-R	GCACCATTCTGCTGGTCTCC

TABLE 2. The primers of Notch signaling target genes used in real-time RT-PCR

HES1-F	GAAGAAAGATAGCTCGCGGCA
HES1-R	CCAGCACACTTGGGTCTGTG
MYC-F	GGTAGTGGAAAACCAGCAGCC
MYC-R	TTCTCCTCCTCGTCGCAGTA
SOX9-F	AGCTCTGGAGACTTCTGAACGA
SOX9-R	GTTCTTCACCGACTTCCTCCG
HEY2-F	GCAACAGGGGGTAAAGGCTAC
HEY2-R	ACCGCGCAACTTCTGTTAGG
GAPDH-F	CCCGCTTCGCTCTCTGCTCC
GAPDH-R	ACCAGGCGCCAATACGACC
